# Supplementary material for: Robust microscale structural superlubricity between graphite and nanostructured surface
Source: Nat Commun. 2023 May 22;14:2931. doi: 10.1038/s41467-023-38680-6 (PMC10202915; doi:10.1038/s41467-023-38680-6)
Supplement: Supplementary file 1 — Supplementary Information [file 41467_2023_38680_MOESM1_ESM.pdf]

Robust microscale structural superlubricity between graphite and nanostructured surface

Xuanyu Huang[1,2,3], Tengfei Li[1,4], Jin Wang[5], Kai Xia[6], Zipei Tan[1,4], Deli Peng[6], Xiaojian Xiang[6], Bin Liu[4], Ming Ma[1,2,3,6*], Quanshui Zheng[1,2,3,4,6*]

[1]Center for Nano and Micro Mechanics, Tsinghua University, Beijing 100084, China;
[2]Department of Mechanical Engineering, Tsinghua University, Beijing 100084, China;
[3]State Key Laboratory of Tribology in Advanced Equipment (SKLT), Tsinghua University, Beijing 10084, China;
[4]Department of Engineering Mechanics, Tsinghua University, Beijing 100084, China;
[5]International School for Advanced Studies, Trieste 34136, Italy.
[6]Institute of Superlubricity Technology, Research Institute of Tsinghua University in Shenzhen, Shenzhen 518057, China;

*Corresponding authors: maming16@tsinghua.edu.cn; zhengqs@tsinghua.edu.cn.

(Supplementary Information)

Content

1. **Fabrication of graphite mesa with Au cap** 3
2. **Fabrication of nanostructured silicon surface.** 4
3. **The transfer process of graphite flakes to the silicon surface.** 10
4. **Statistical analysis of nanostructures on silicon** 12
5. **Friction measurement based on AFM system** 15
6. **Interface characterization method for tribological experiment** 17
7. **The wear between graphite flake and atomic flat silicon surface** 20
8. **The setup of finite element simulation of edge warping mechanism** 23
   8.1 Van der Waals interaction in the finite element methods 23
   8.2 Mesh convergence analysis 25
   8.3 Simulation of graphite flake pressed on flat silicon surface 26
   8.4 Calculation of the real contact area between graphite flakes and nanostructured silicon 27
   8.5 Simulation of graphite flake pressed on nanostructured silicon surface with difference separation. 29
9. **Layer-number-dependent molecular dynamics simulations of adhesion at the graphene/silicon interface** 32
10. **Analyse of the water film at the nanostructured surface** 33

Supplementary Table 3 34

11. **General surface modification methods for superlubric generators (SLGs) application scenarios** 34

1. Fabrication of graphite mesa with Au cap

We fabricated square arrays of graphite mesas with an Au cap on highly ordered pyrolytic graphite (HOPG), specifically of ZYB grade (Brucker)[1]. The detailed fabrication process is depicted in Supplementary Fig. 1a. Initially, a double layer of photoresist consisting of LOR 1A (100 nm) and ZEP (400 nm) was spun onto the freshly cleaved surface of the HOPG, as illustrated in Supplementary Fig. 1a (i). Next, the photoresist in the region corresponding to the mesa pattern array was selectively removed through an electron beam lithography and development process, as depicted in Supplementary Fig. 1a (ii). Subsequently, an Au film with a thickness of 100 nm was deposited onto the surface via electron beam evaporation, as shown in Supplementary Fig. 1a (iii). By employing a lift-off process, the Au pattern array was obtained, as illustrated in Supplementary Fig. 1a (iv). Finally, utilizing the Au pattern array as a mask, we achieved the formation of graphite mesas with an Au cap through an oxygen reactive ion etching process, as shown in Supplementary Fig. 1a (v). The etching depth was measured to be 0.8 μm.

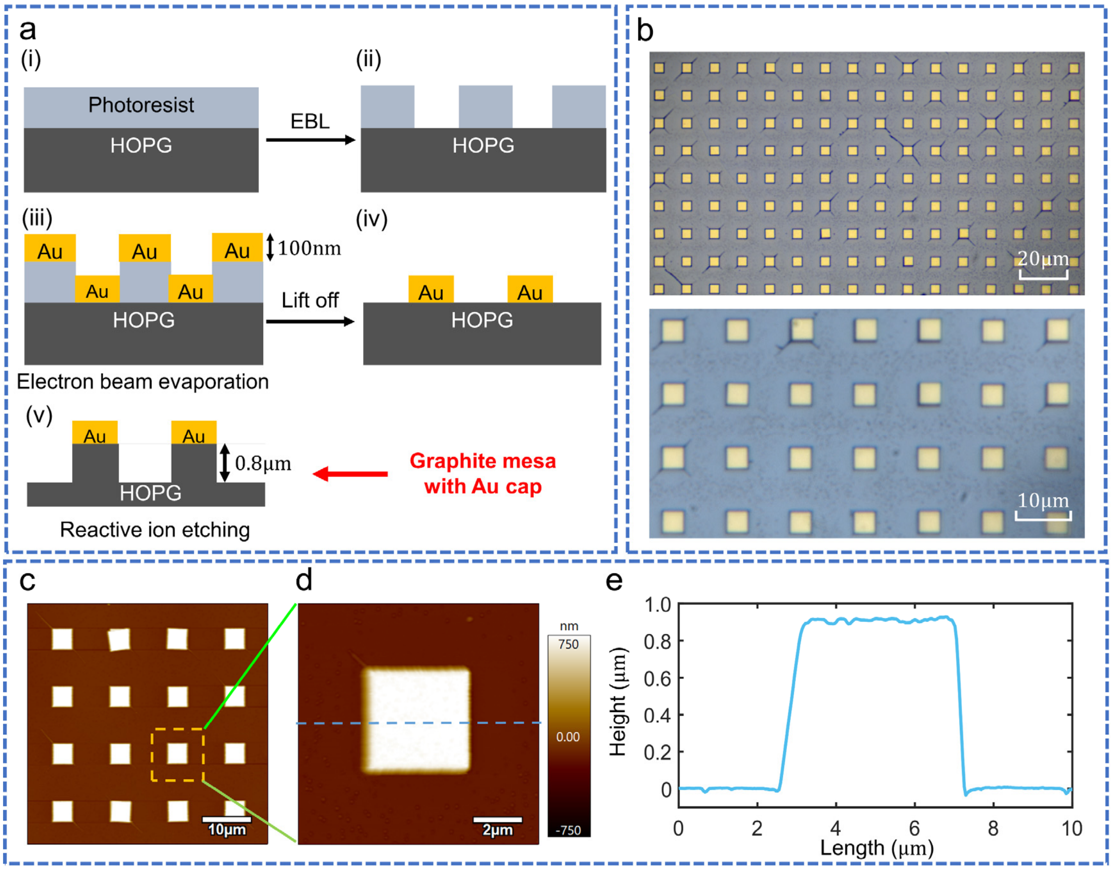

Supplementary Fig. 1 Preparation of graphite mesa with Au cap. a Fabrication process of graphite mesa: (i) Spin-coating double layer photoresist LOR 1A (100 nm)/ZEP (400 nm) on the freshly cleaved surface of HOPG. (ii) Electron beam lithography. (iii) Electron beam evaporation of a layer of Au film. (iv) Lift off to get the Au pattern array. (v) Oxygen reactive ion etching. **b** Optical observation of fabricated graphite mesa with Au cap, where the upper and lower image are 20X and 50X lens observation respectively. **c** Atomic force microscope (AFM) morphology of fabricated graphite mesa with Au cap. **d** Local morphology of a single graphite mesa in the orange dashed region in **c**. **e** Section view of the blue dashed line in **d**, the height of the graphite mesa is around 913 nm.

In order to show the effect of the fabricated graphite mesa with Au cap, we carried out a series of characterizations. The observations of fabricated graphite mesa with Au film validated by optical microscopy (HiRox KH-3000) are shown in Supplementary Fig. 1b, where the upper and lower image are 20X and 50X lens observation respectively, and the brighter region in the images is the protruding graphite mesa with Au cap. The atomic force microscope morphology image of 4×4 graphite mesa array with Au cap is shown in Supplementary Fig. 1c, where the local morphology of a single graphite mesa in the orange dashed region is shown in Supplementary Fig. 1d. Supplementary Fig. 1e is the section view of the blue dashed line in Supplementary Fig. 1d, and the height of the graphite mesa is around 913 nm.

2. Fabrication of nanostructured silicon surface.

In order to reproducibly fabricate uniform nanostructures on silicon surface, we produced a fabrication process as shown in Supplementary Fig. 2a. Firstly, we ultrasonically clean the silicon surface with acetone, alcohol and deionized water for 5 minutes, and then perform the surface hydrophilic treatment by oxygen plasma, as shown in Supplementary Fig. 2a (i). Then mix 200 nm polystyrene (PS) microsphere solution (mass fraction 10%, produced by Huge Biotechnology Company) with alcohol

and deionized water in a ratio of 1:2:2 and ultrasonic for 30 minutes to obtain the diluted solution. Further immerse the silicon surface with deionized water and use the pipette to drop the diluted solution 250 μL, and make it self-assemble on the liquid interface, as shown in Supplementary Fig. 2a (ii).

Secondly, we evaporate the deionized water in atmospheric environment, and deposit the self-assembled PS microsphere array on the silicon surface, as shown in Supplementary Fig. 2a (iii). Then, we reduce the size of PS microspheres by oxygen plasma etching (power: 400 W, oxygen flow: 400 sccm, etching time: 4 min), as shown in Supplementary Fig. 2a (iv). In order to control the geometric size of the fabricated nanostructures, we need to accurately control the size and spacing of the PS microsphere array used as the mask, so we performed the scanning electron microscope (SEM) observation during the oxygen plasma etching process of the PS microspheres. Supplementary Figs. 2b-d show the SEM observations of the PS microsphere array deposition, oxygen plasma etching for 3 min and 5min, respectively, and the multiples gradually increase from left to right of each image. It can be seen that the microspheres are tightly arranged on the silicon surface when they are initially self-assembled (Supplementary Fig. 2b). After 3 minutes of oxygen plasma etching, the size of the PS microsphere array was reduced from 200nm to around 150nm (Supplementary Fig. 2c). When the etching time was further extended to 5 minutes, the size of the PS microsphere array was reduced to around 100 nm (Supplementary Fig. 2d).

Thirdly, we use PS microsphere array as mask to obtain the nanostructures by ion beam etching process (beam: $1\ \mathrm{mA/cm^2}$, energy: 500 eV, etching time: 15s), as shown in Supplementary Fig. 2a (v).

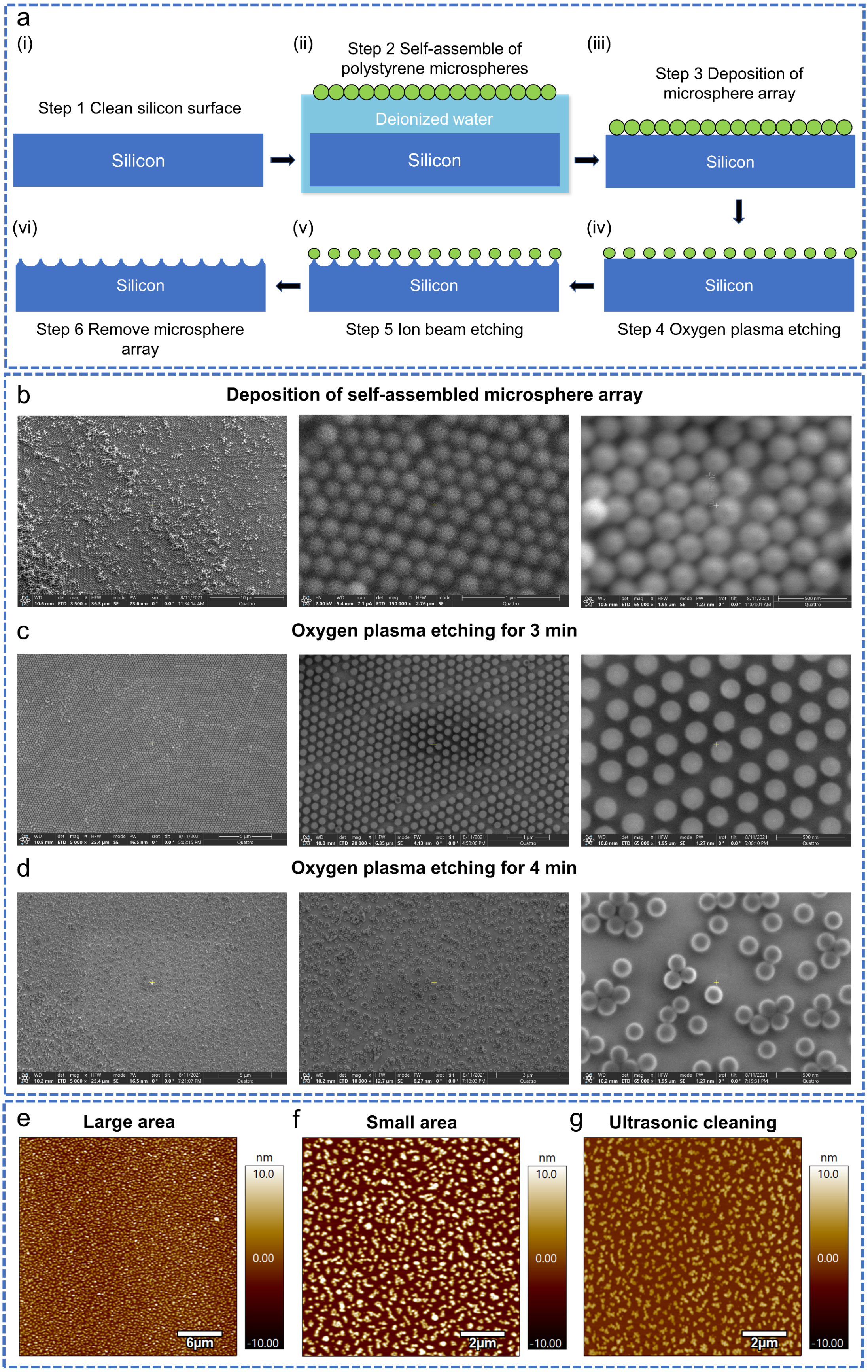
a
(i)
Step 1 Clean silicon surface
Silicon
(ii)
Step 2 Self-assemble of polystyrene microspheres
Deionized water
Silicon
(iii)
Step 3 Deposition of microsphere array
Silicon
(iv)
Silicon
Step 4 Oxygen plasma etching
(v)
Silicon
Step 5 Ion beam etching
(vi)
Silicon
Step 6 Remove microsphere array
b
Deposition of self-assembled microsphere array
c
Oxygen plasma etching for 3 min
d
Oxygen plasma etching for 4 min
e
Large area
nm
10.0
0.00
-10.00
6μm
f
Small area
nm
10.0
0.00
-10.00
2μm
g
Ultrasonic cleaning
nm
10.0
0.00
-10.00
2μm

Supplementary Fig. 2 Fabrication process of nanostructured silicon surface and its characterizations. a Fabrication process of nanostructures on silicon surface: (i) Step 1: Clean the silicon surface. (ii) Step 2: Submerge the silicon surface with deionized water and let the polystyrene (PS) microspheres self-assemble on the liquid interface. (iii) Step 3: Evaporate the deionized water, and then deposit the self-assembled PS microsphere array on the silicon surface. (iv) Step 4: Oxygen plasma etching to reduce the size of PS microspheres. (v) Step 5: Ion beam etching. (vi) Step 6: Remove the PS microsphere array. **b** Scanning electron microscope (SEM) observation of self-assembled PS microsphere array. **c** and **d** are the SEM observation after 3min and 5min of oxygen plasma etching, respectively. The multiples gradually increase from left to right of each image in **b-d**. **e** and **f** are the large area and small area atomic force microscope (AFM) morphologies of the fabricated nanostructured silicon surface after removing PS microsphere array. **g** AFM morphology of the fabricated nanostructured after final ultrasonic cleaning.

Lastly, we remove the PS microsphere array by excessive oxygen plasma etching, as shown in Supplementary Fig. 2a (vi). The large area and small area atomic force microscope (AFM) morphologies of the fabricated nanostructured silicon surface after removing PS microsphere array are shown in Supplementary Figs. 2e and f respectively. It can be observed that there is still some dirt remaining on the nanostructure. Then we use acetone, alcohol, and deionized water ultrasonic cleaning to remove the residual solvents and polystyrene spheres on the nanostructures, and the AFM morphology of the fabricated nanostructured after final ultrasonic cleaning is shown in Supplementary Fig. 2g. The nanostructures are uniformly distributed, and the peak height is uniform.

To demonstrate that solvents and polystyrene spheres were completely expelled after the fabrication of the nanostructured surface and exclude the transfer of polystyrene to the silicon surface resulting in different tribological conditions, we used a surface elemental analysis method with a shallower detection depth, that is, X-ray photoelectron spectroscopy (XPS, Thermo Fisher, Model: ESCALAB 250Xi, Source

gun type: Al K Alpha, Spot size: 500 µm, Lens mode: Standard, Pass energy 30.0 eV) to characterize the prepared nanostructured silicon surface, as shown in Supplementary Fig. 3. Supplementary Fig. 3a shows the results of the full-scan spectra (Energy step size: 1.000 eV), where the three characteristic elements of interest (*C1s*, *O1s* and *Si2p*) can be seen. Further, we carried out narrow scan spectra (Energy step size: 0.050 eV) for the three characteristic peaks, as shown in Supplementary Figs. 3b-d respectively. First of all, according to the narrow scan spectra results of *C1s* in Supplementary Fig. 3b we measured, the peak energy is 284.82 eV, which corresponds to the adventitious hydrocarbon contamination (C aromatic and aliphatic)[2]. Compared with the standard *C1s* XPS spectrum features of polystyrene (Supplementary Table 1)[3], we found that the peak at 291.27 eV corresponding to the shake-up transition does not appear in our measurement results[3,4], which indicates that no polystyrene remains on the nanostructured silicon surface.

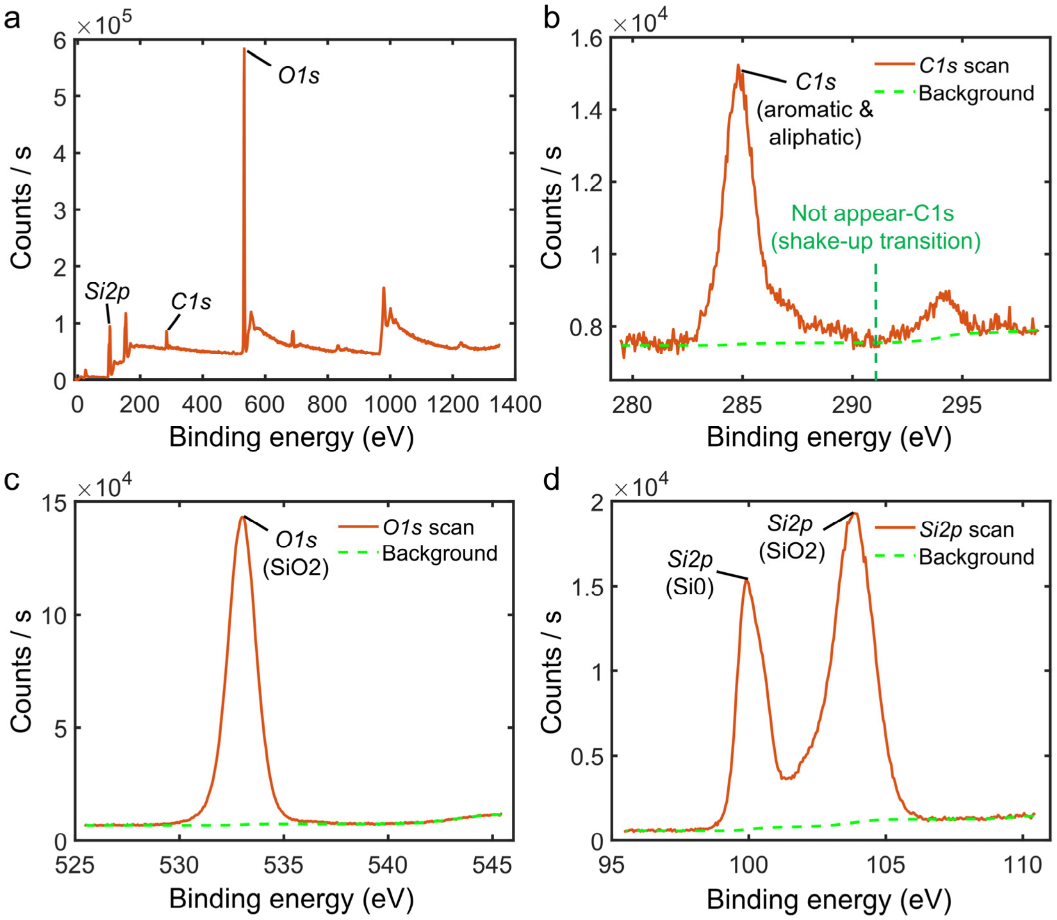

Supplementary Fig. 3 X-ray photoelectron spectroscopy (XPS) characterization of prepared nanostructures on silicon surface. a Full-scan spectra (Energy step size: 1.000 eV). **b-d** are the narrow scan spectra (Energy step size: 0.050 eV) for the three characteristic peaks of *C1s*, *O1s* and *Si2p* respectively.

In addition, there are two peaks in the narrow scan spectra of *Si2p* (Supplementary Fig. 3d), which are correspond to the elemental silicon and the tetravalent silicon (surface oxide layer)[2], respectively, while the peak of *O1s* in Supplementary Fig. 3b corresponds to the lattice oxygen in the surface oxide layer. More detailed data about the above elements and characteristic peaks are shown in Supplementary Table 2[2].

Supplementary Table 1 Standard spectral features of polystyrene[3].

Element/ Transition	Peak Energy (eV)	Peak Width FWHM (eV)	Peak Area (eV×Cts/s)	Sensitivity Factor	Concentration (at. %)	Peak Assignment
C1s	284.72	1.31	285262	1	71.4	C aromatic
C1s	285.00	1.59	87143	1	21.8	C aliphatic
C1s	291.27	1.13	27011	1	6.8	shake-up transition

Supplementary Table 2 XPS peak table of prepared nanostructured surface.

Element/ Transition	Peak Energy (eV)	Peak Width FWHM (eV)	Peak Area (eV×Cts/s)	Sensitivity Factor	Concentration (at. %)	Peak Assignment
Si2p	100.16	1.20	18257.76	0.9	11.80	Si0
Si2p	103.71	1.95	36203.67	0.9	23.42	SiO2
C1s	284.82	1.46	15450.75	1	9.44	Hydrocarbon contamination
O1s	533.00	1.55	239687.62	2.881	55.34	SiO2

3. The transfer process of graphite flakes to the silicon surface.

After the fabrication process of graphite mesa, we need to select a graphite flake with single crystal superlubric interface and transfer to the fabricated silicon surface, and the specific process is shown in Supplementary Fig. 4, where the left and right side of each image are schematic diagram and optical microscope observation respectively. Firstly, we utilized a tungsten microtip, controlled by a micromanipulator (Kleindiek MM3A), to attach the Au cap of the graphite mesa fabricated as described in Supplementary Fig. 1. This process is illustrated in Supplementary Fig. 4a. By applying shear stress using the micromanipulator, we achieved a controlled splitting of the graphite mesa at approximately 2 μm from the vertical direction, as shown in Supplementary Fig. 4b. Next, we removed the microtip to observe whether the sheared graphite flake underwent self-recovery motion (SRM)[5], which would indicate the presence of a single crystal structural superlubric bottom surface[6]. This observation is depicted in Supplementary Fig. 4c. Subsequently, we re-attached the graphite flake, which exhibited SRM properties, using the microtip and completed the splitting process to completely separate the flake. We then picked up the dangling graphite flake, which was being dragged by the microtip, as shown in Supplementary Figs. 4d and e. Finally, we carefully placed the dangling graphite flake onto the fabricated silicon surface using the micromanipulator. Due to the stronger adsorption force between the graphite flake and silicon compared to that between the microtip and graphite flake, the graphite flake remained on the silicon surface, as depicted in Supplementary Fig. 4f. This process resulted in the formation of the graphite flake/silicon interface shown in Fig. 1a. Given the absence of exposure to the environment throughout the entire fabrication process, as depicted in Supplementary Fig. 1a, the cleaved interface of the ultimately transferred graphite flake remained uncontaminated.

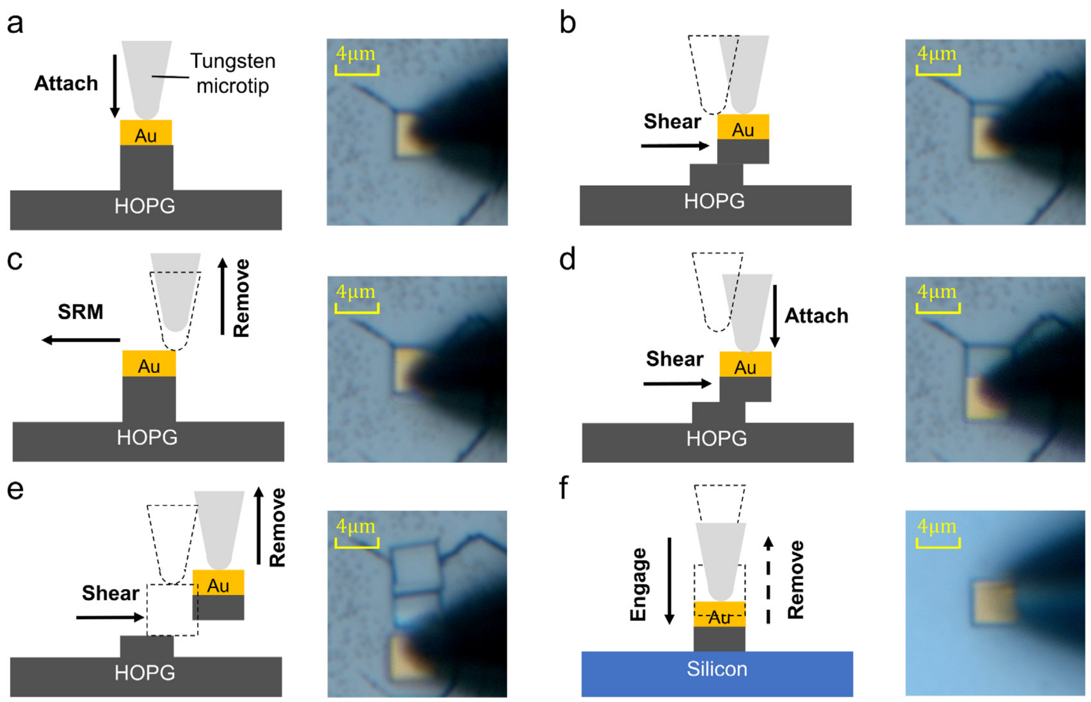

Supplementary Fig. 4 Transfer process of graphite flake and its optical observation. a Attach a tungsten microtip with a tip curvature radius of around 2 μm to the graphite mesa. **b** Apply a shear stress by the micromanipulator to split the graphite mesa a distance of ~2 μm from the vertical direction. **c** Remove the microtip to observe whether the self-recovery motion (SRM) has occurred. **d** Attach the microtip again same as **b** and split the graphite mesa out completely. **e** Pick up the dangling graphite flake dragged by the microtip. **f** Place the graphite flake slowly by micromanipulator on the fabricated silicon surface.

After completing the transfer process of graphite flakes to nanostructured silicon surface, we used scanning electron microscopy (SEM) to characterize the transferred interface. Supplementary Fig. 5a shows a large-view SEM observation of the graphite flake transferred on the nanostructured silicon, and Supplementary Fig. 5b shows a partial zoom-in SEM observation of the green dotted frame area in Supplementary Fig. 5a, where the square brighter region in the middle is the graphite flake, and the dark spots around graphite flake are the nano rough peaks.

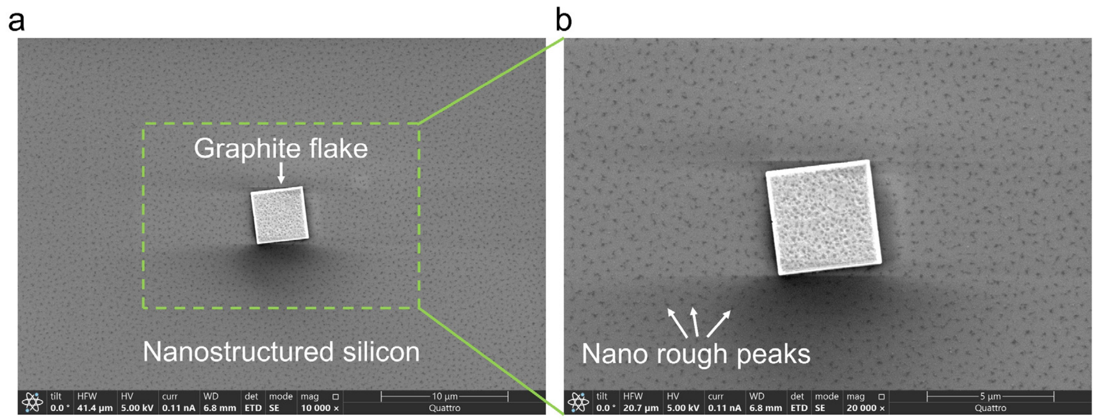

Supplementary Fig. 5 Scanning electron microscopy (SEM) observation of graphite/nanostructured silicon interface. **a** Large-view SEM observation of the graphite flake transferred on the nanostructured silicon. **b** Partial zoom-in SEM observation of the green dotted frame area in **a**.

4. Statistical analysis of nanostructures on silicon

In order to obtain the geometric characteristics of the fabricated nanostructures and provide a basis for subsequent simulation, we designed the following algorithm to identify the location of rough peaks through AFM morphology data and count the average height h_{peak} and spacing d_{peak} of the rough peaks. Firstly, we import the AFM morphology data of fabricated nanostructured silicon surface, as shown in Supplementary Fig. 6a. Then, we count the 256×256 data points in Supplementary Fig. 6a to get the statistics histogram as shown in Supplementary Fig. 6b. It can be seen that the highest frequency corresponds to the height of the substrate with -2.5 nm.

Secondly, we get the binarization image base on Supplementary Fig. 6a with the threshold of 1 nm, as shown in Supplementary Fig. 6c. In order to locate the rough peak more accurately, we perform 4-connected erosion base on Supplementary Fig. 6c to obtain the erosion image as shown in Supplementary Fig. 6d.

Thirdly, we search for all the single connected domains in Supplementary Fig. 6d, and each domain corresponds to a rough peak. Next, we will capture the specific location and average height of each rough peak as following method. Take the i-th

connected domain as an example, suppose the coordinates and height of the data points contained in it are $\left(x_{\mathrm{j}}^{(\mathrm{i})}, y_{\mathrm{j}}^{(\mathrm{i})}\right)$ and $h_{\mathrm{j}}^{(\mathrm{i})}$ $(j = 1,2,3, \dots, N)$ respectively. We can calculate the center coordinates $\left(x_{\mathrm{c}}^{(\mathrm{i})}, y_{\mathrm{c}}^{(\mathrm{i})}\right)$ and average height $h_{\mathrm{peak}}^{(\mathrm{i})}$ of the i-th rough peak by

$$x_{\mathrm{c}}^{(\mathrm{i})} = \frac{\sum_{j=1}^{N} x_{\mathrm{j}}^{(\mathrm{i})} h_{\mathrm{j}}^{(\mathrm{i})}}{\sum_{j=1}^{N} h_{\mathrm{j}}^{(\mathrm{i})}},$$
$$y_{\mathrm{c}}^{(\mathrm{i})} = \frac{\sum_{j=1}^{N} y_{\mathrm{j}}^{(\mathrm{i})} h_{\mathrm{j}}^{(\mathrm{i})}}{\sum_{j=1}^{N} h_{\mathrm{j}}^{(\mathrm{i})}}, \tag{S1}$$
$$h_{\mathrm{peak}}^{(\mathrm{i})} = \frac{\sum_{j=1}^{N} h_{\mathrm{j}}^{(\mathrm{i})}}{N} + 2.5,$$

where the height calculation considers the substrate height. The positions of all captured rough peaks $\left(x_{\mathrm{c}}^{(\mathrm{i})}, y_{\mathrm{c}}^{(\mathrm{i})}\right)$ are marked with blue dots in Supplementary Fig. 6e.

Finally, we calculate the distance of the nearest rough peak for each rough peak $d_{\mathrm{peak}}^{(\mathrm{i})}$, and then obtain the statistical histogram as shown in the upper part of Supplementary Fig. 6f. The statistical histogram of average height $h_{\mathrm{peak}}^{(\mathrm{i})}$ is also plotted in the lower part of Supplementary Fig. 6f. It can be seen that the rough peak spacing d_{peak} and height h_{peak} corresponding to the highest frequency are $0.354\ \mu\mathrm{m}$ and $6.82\ \mathrm{nm}$ respectively, which are slightly different from the 200 nm diameter of the nanospheres we predicted and the designed etching height of 7 nm. The former is mainly due to the movement of PS microspheres by the gas flow in the later stage of the oxygen plasma etching process, as shown in Supplementary Fig. 2d, and the latter is mainly due to the slow etching rate at the beginning of ion beam etching process.

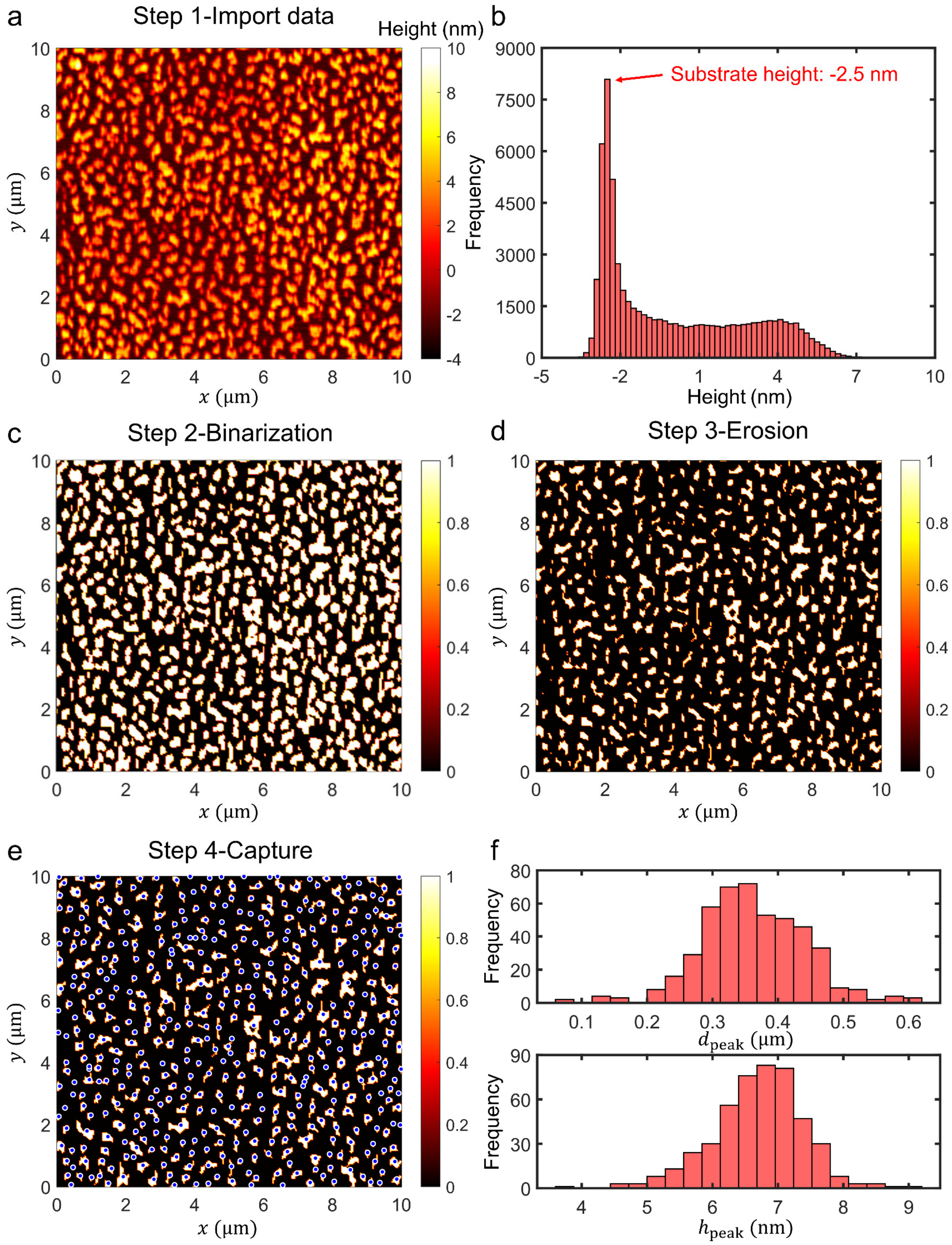

Supplementary Fig. 6 Statistical analysis of nanostructures on silicon. a Import the AFM morphology data. **b** Statistics histogram of all height data points in **a**, where the substrate height is around -2.5 nm. **c** Binarized image obtained by **a** with threshold of 1nm. **d** Four-connected domain erosion image obtained by **c**. **e** Capture the location and average height of each rough peak by searching the data information of each single

connected domain in **d**. **f** Statistics histogram of rough peak spacing d_{peak} (upper) and height h_{peak} (lower).

Furthermore, we determine the shape of the rough peak to provide a basis for subsequent simulation modeling. We select a general rough peak and export its height data as shown in Supplementary Fig. 7 (the blue square points). Then, we use the function $y = A\sin\left(\frac{\pi x}{L}\right)^n$ to fit the data points, where $A = 7\ \text{nm}, L = 350\ \text{nm}$, the accurately fitted n is 10.22. In order to facilitate subsequent simulation modeling, we rounded $n = 10$ to get the fitted curve as shown in Supplementary Fig. 7 (red solid line).

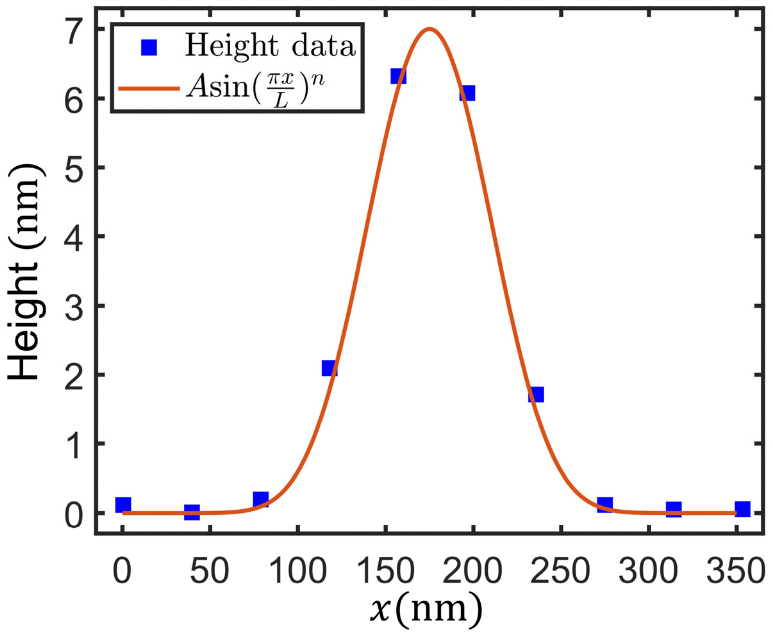

Supplementary Fig. 7 Fitting of rough peak shape. The blue square points are the height data of a general rough peak, and the red solid line is the curve fitted with the function $y = A\sin\left(\frac{\pi x}{L}\right)^n$, where $A = 7\ \text{nm}, L = 350\ \text{nm}$ and $n = 10$ (Accurately fitted n is 10.22, the figure shows the result of rounding $n = 10$).

5. Friction measurement based on AFM system

Friction measurements of the graphite/n-Si heterostructures were conducted in an ambient atmosphere at a temperature of 25 ± 1 °C and a relative humidity of 30 ± 4%. The experimental setup consisted of a commercial NTEGRA upright AFM (Cypher S-Oxford Instruments), a XYZ piezoelectric displacement platform, a high numerical aperture objective lens (×20), and a visualized AFM tip (ACCESS-NC-GG (Appnano)). A schematic of the experimental setup is presented in Fig. 1a. To ensure precision, we carefully applied pressure to the Au cap of the graphite flake using the AFM tip, employing both the optical microscope and the piezoelectric displacement platform. The in-situ calibration of the AFM tip was performed using the Sader method[7,8] for measuring normal direction forces and the diamagnetic levitation spring system[9] for measuring lateral direction forces. The silicon's bottom surface is securely affixed to the piezoelectric stage using tape. A photodiode captures the reflection of a laser beam projected onto the cantilever of the AFM tip, enabling precise measurement of lateral force during the sliding process by tracking the movement of the laser spot resulting from cantilever deformation.

Take the friction measurement in Fig. 2a of main text as an example, the normal force F_{N} was applied by using the AFM cantilever, which can be written as

$$F_{\mathrm{N}} = S_{\mathrm{n}} k_{\mathrm{n}} V_{\mathrm{n}}, \tag{S2}$$

where S_{n} is the optical lever sensitivity, k_{n} is the normal spring constant, and V_{n} is optical detector signal. The lever sensitivity is measured by performing a standard force curve measurement as shown in Supplementary Fig. 8a, which includes loading (Forward, blue line) and unloading (Backward, red line) process. We also obtained average slope $S_{\mathrm{n}}^{(\mathrm{av})} = 87.67\ \mathrm{nm/V}$ through linear fitting. The normal spring constant $k_{\mathrm{n}} = 27.86\ \mathrm{N/m}$ was calibrated by the Sader method [7]. The optical detector signal $V_{\mathrm{n}} = 6\ \mathrm{V}$ was set by AFM instrument. Therefore, we calculated the normal force of $F_{\mathrm{N}} = 14.65\ \mu\mathrm{N}$.

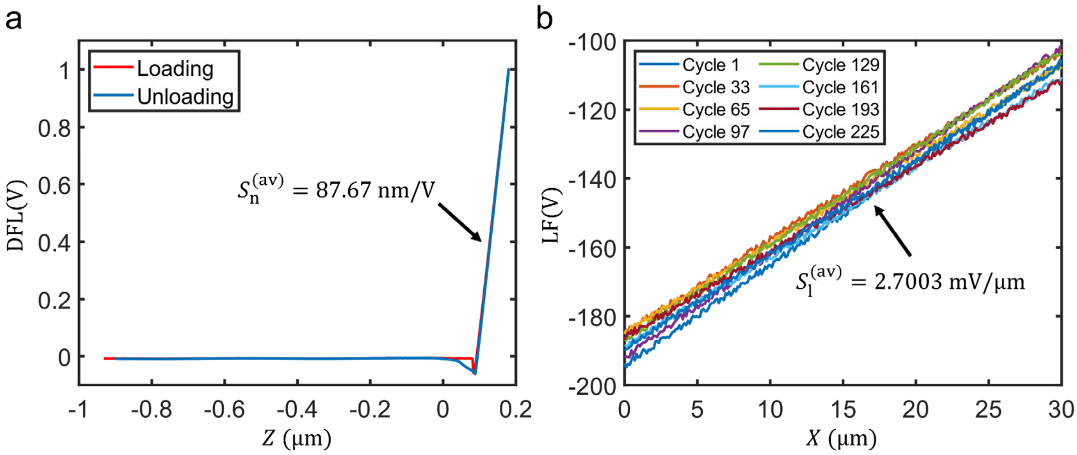

Supplementary Fig. 8 Lever sensitivity measurement for normal and lateral calibration: (a) Standard force curve measurement for normal calibration, which includes the forward (blue) and backward (red) process. (b) Diamagnetic lateral force curve measurement for lateral calibration, where different colours represent different test lines.

Similar to the normal force, the friction force f was measured by AFM cantilever, which can be written as

$$f = \frac{k_l}{S_l} \times V_l, \tag{S3}$$

where S_l is the optical lever sensitivity, k_l is the lateral spring constant of diamagnetic levitation system[9], and V_l is measured frictional optical detector signal [9]. The $k_l = 5.26 \times 10^{-2}$ N/m was calibrated by using high-speed CCD to detect the vibration frequency of the levitate graphite sheet and precision balance to measure its mass[9]. The optical lever sensitivity was measured by using the AFM tip to drag the levitate graphite sheet and measure its lateral force curve as shown in Supplementary Fig. 8b, we tested 256 times, and 16 of them are drawn in the figure, and we obtained its average slope $S_l^{(av)} = 2.7003$ mV/μm through linear fitting. Therefore, we calculated the lateral force coefficient $\alpha = k_l/S_l = 19.48$ nN/mV.

6. Interface characterization method for tribological experiment

Here, we provide a comprehensive description of the experimental process and methods, as illustrated in Fig. 2, Fig. 5, and Supplementary Fig. 10. Take Fig. 5 of the main text as an example, we first test the morphology of silicon sliding interface before and after friction test based on the atomic force microscope system (Cypher S-Oxford Instruments). Supplementary Fig. 9 gives a schematic diagram of the operation flow, the yellow solid block represents the graphite flake and the blue solid block represent the silicon substrate at the bottom, which includes the following steps:

- Step 1: We first find the transferred graphite flake on the silicon surface through the optical microscope as shown in Supplementary Fig. 9a, and then we measure the morphology of the right area 10 μm away from the center of the graphite flake based on a piezoelectric localization system, as shown in Supplementary Fig. 9b.
- Step 2: We use the AFM tip (ACCESS-NC-GG(Appnano)) to apply normal load on the graphite flake, and then move it to the right by 10 μm to the center of the previous morphological characterization region, as shown in Supplementary Fig. 9c.
- Step 3: Then we use the AFM tip to continue to drag the graphite flake to slid on the silicon surface to measure the friction force in situ, as shown in Supplementary Fig. 9d.
- Step 4: After the sliding process, we use the AFM tip to apply normal load on the graphite flake, and then move it to the left by 10 μm out of the characterization region, as shown in Supplementary Fig. 9e.
- Step 5: Then, we measure the morphology of the right area 10 μm away from the center of the graphite flake again after the sliding process, which contain the sliding region, as shown in Supplementary Fig. 9f.

After completing the above test process, we can continue to use the graphite flake as a mark to perform other characterization on the sliding region, such as friction mapping and Raman mapping characterization.

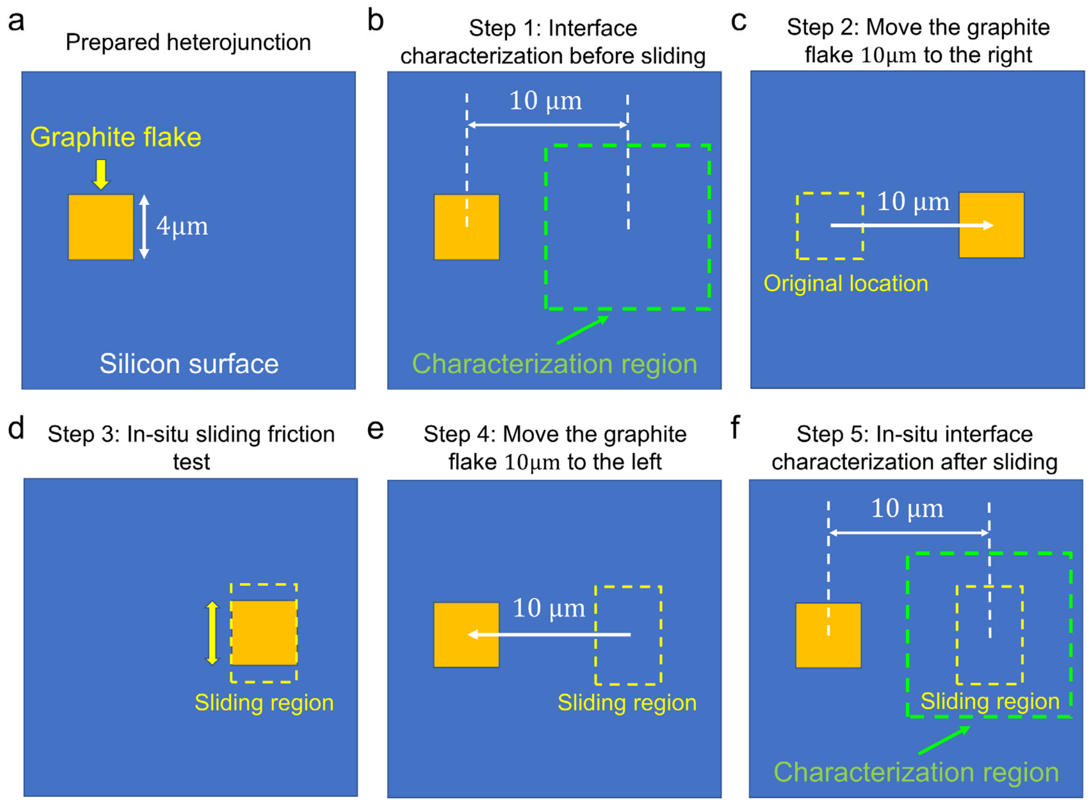

Supplementary Fig. 9 Interface ccharacterization process of slid silicon based on atomic force microscope system. **a** Prepared graphite flake/silicon heterojunction. **b** Step 1: Characterize the morphology $10\mu m \times 10\mu m$ silicon region centered at $10\mu m$ on the right side of the graphite flake before sliding test. **c** Step 2: Move the graphite flake $10\mu m$ to the right to the center of the characterization region. **d** Step 3: In-situ sliding friction test. **e** Step 4: Move the graphite flake $10\mu m$ to the left to out of the characterization region. **f** Step 5: Characterize the morphology $10\mu m \times 10\mu m$ silicon region in-situ after sliding test, which includes the sliding region.

After completing all the characterizations of slid silicon interface, we need to characterize the slid graphite flake interface to determine whether it occurred wear. To achieve this, we employ AB glue to lift the graphite flake, effectively overcoming the van der Waals adsorption force between the graphite flake and the silicon interface. For a comprehensive understanding of this process, please refer to the relevant references[10,11]. Subsequently, we invert the microtip by 180 degrees to enable optical microscope observations of the slid graphite flake interface, as exemplified in Fig. 2j,

Fig. 5i of the main text, and Supplementary Fig. 10j. Moreover, Raman characterization can be performed on the inverted slid graphite flake interface to determine the presence of any discernible damage, indicated by the presence of a *D* peak ($1350\ \mathrm{cm}^{-1}$).

7. The wear between graphite flake and atomic flat silicon surface

In order to understand the phenomenon of the large friction between the graphite flake and the atomic-level flat silicon surface in Fig. 1d of main text, we performed detailed in-situ characterizations of the sliding interface (See Methods Section and Supplementary Section 6 for details). First of all, we use the same experimental set-up as Fig. 1a of main text under the same environmental conditions and drag the $4 \times 4\ \mu\mathrm{m}$ graphite flake with the AFM tip to slide on the atomic-level silicon surface for 1024 cycles with the normal load of $20.04\ \mu\mathrm{N}$ and the speed of $6\ \mu\mathrm{m/s}$. Measured sliding friction force as shown in Supplementary Fig. 10a is always maintained at a large level (> 5μN) throughout the process.

Secondly, we tested the $10\ \mu\mathrm{m} \times 10\ \mu\mathrm{m}$ topography of the sliding region in situ as shown in Supplementary Fig. 10b, where the white highlighted region is the graphite flake, and the yellow dash frame is the boundary of the sliding region on the silicon surface. The partial enlarged view of the upper boundary in Supplementary Fig. 10b is shown in Supplementary Fig. 11a.

Thirdly, we use the AFM tip to drag the graphite flake $6\ \mu\mathrm{m}$ to the left out of sliding region (Supplementary Fig. 11b), and then continue to characterize the topography in situ as shown in Supplementary Fig. 10c, where the yellow dash frame is the boundary of the sliding region. There were debris on the boundary of the sliding region, but the middle topography of the sliding region as shown in Supplementary Fig. 10d is still atomically flat without any wear. We further performed the in-situ friction mapping characterization between the AFM tip and the slid silicon surface, as shown in Supplementary Fig. 11c, where there is a high friction force distribution on the boundary of the sliding region. Both the inside and outside of the sliding region have the similar friction magnitudes. Therefore, based on the above characterization results,

the wear mainly occurred at the edge of the contact region instead of internal contact region.

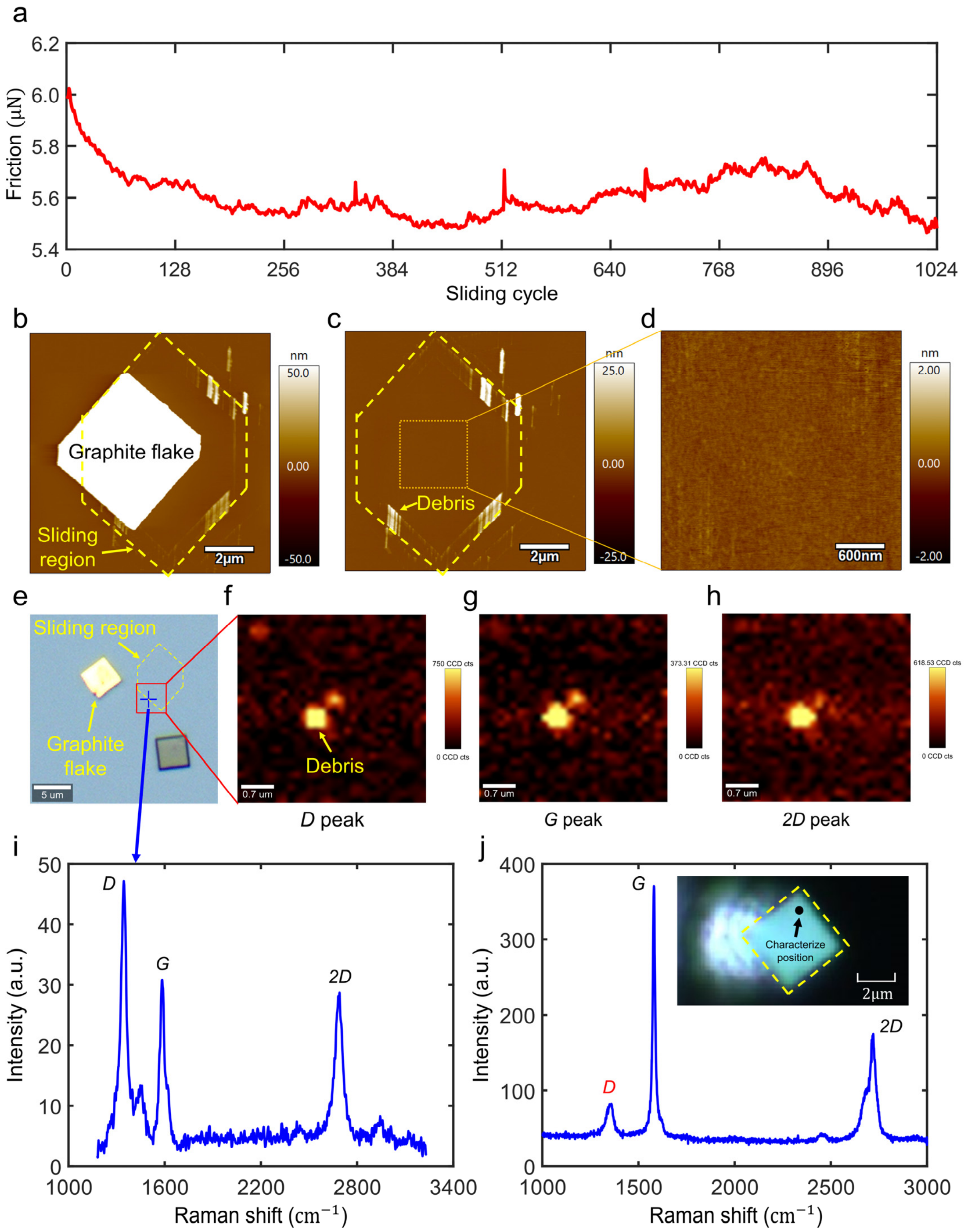

Supplementary Fig. 10 Characterization of wear between graphite flake and atomic flat silicon surface. **a** Measured friction force during 1024 continuous sliding cycles with a displacement amplitude of 3 μm and speed of 6 μm/s under a normal force of $F_{\mathrm{N}} = 20.04$ μN. **b** Topography (10 μm × 10 μm) of the atomic-level smooth silicon surface in situ after 1024 sliding cycles, where the white highlighted region is

the graphite flake, and the yellow dashed frame is the boundary of the sliding region. **c** Topography (10 μm × 10 μm) obtained after using AFM tip to drag the graphite flake 6 μm to the left out of sliding region and then offset 2 μm to the right, there is wear debris on the edge of the sliding region. **d** Local topography (3 μm × 3 μm) of the middle area in sliding region (orange dashed frame in **c**). **e-j** are the Raman characterization results of the sliding silicon region, where **e** is the optical observation, the red area is the scan area of 4 μm × 4 μm (including wear debris), **f-h** are the intensity distributions of *D* peak (1350 cm^{-1}), *G* peak (1580 cm^{-1}) and *2D* peak (2700 cm^{-1}), respectively, there is obvious graphite composition at the position of wear debris. **i** Single-point Raman spectrum at the position of wear debris on slid silicon surface (blue cross mark in **e**), there are obvious *D*, *G* and *2D* graphite peaks. **j** Single-point Raman spectrum at the critical position (black dot in illustration) on slid graphite interface, there is obvious *D* peak indicating defects caused by wear, where the illustration is the optical observation of flipped graphite flake.

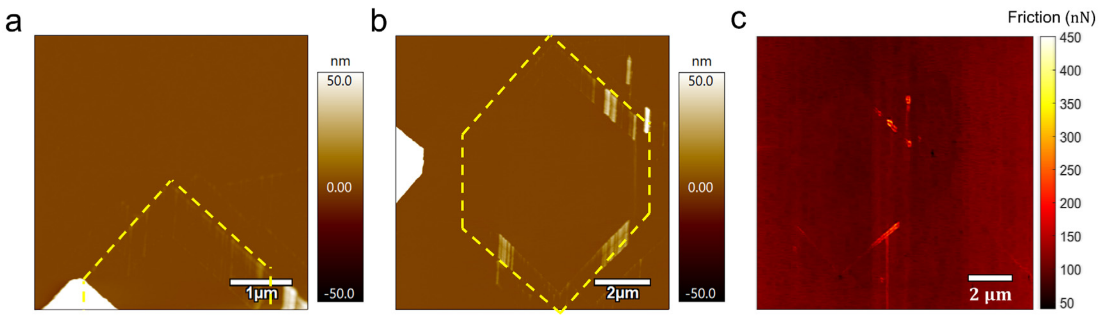

Supplementary Fig. 11 The additional surface characterization results base on the friction test of Supplementary Fig. 10. a Partial enlarged scan of Supplementary Fig. 10b. **b** Move the graphite flake to the left by 6μm based on Supplementary Fig. 10b. **c** Friction mapping characterization in-situ from Supplementary Fig. 10c, where the normal force applied by the AFM tip is 1 μN, and the sliding frequency is 1 Hz.

Lastly, in order to clarify the composition of wear debris, we did the Raman mapping characterization on the slid silicon surface. Supplementary Fig. 10e is the microscope observation, where the red frame area is the Raman characterization area,

which contains the wear debris at the boundary of sliding region (yellow dash frame). The intensity distributions of *D* peak (1350 $\mathrm{cm^{-1}}$), *G* peak (1580 $\mathrm{cm^{-1}}$) and *2D* peak (2700 $\mathrm{cm^{-1}}$) are shown in Supplementary Figs. 10f-h, respectively, there is large intensity at the position of the wear debris in three images. The single-point Raman spectrum at the high intensity position in Supplementary Figs. 10f-h (the blue cross mark in Supplementary Fig. 10e) is shown in Supplementary Fig. 10i, where contains the obvious *D*, *G* and *2D* graphite peaks, which indicates that the wear debris contains graphite. We also performed Raman characterization at the critical position (black dot in illustration) on the slid graphite flake interface, as shown in Fig. 10j. The obvious *D* peak on the slid graphite flake interface indicates that there was wear to the graphite flake interface after 1024 sliding cycles. Combining all the Raman characterization results above, we can conclude that the carbon atoms along the edge of the graphite flake are dragged off to the substrate through the strong chemical bond interaction with silicon.

8. The setup of finite element simulation of edge warping mechanism

8.1 Van der Waals interaction in the finite element methods

The Van der Waals (vdW) interaction between the bottom surface of the graphite flake and the top surface of the nanostructured silicon substrate in the finite element methods is derived from the Lennard-Jones (LJ) potential, which is widely used to describe vdW forces between materials. The LJ potential between carbon and silicon atoms is given by:

$$V_{\mathrm{LJ}}(r) = 4\varepsilon\left[\left(\frac{\sigma}{r}\right)^{12} - \left(\frac{\sigma}{r}\right)^{6}\right], \tag{S4}$$

where $\sigma = 3.629$ Å, and $\varepsilon = 0.205$ kcal/mol[12].

For atoms in two surfaces in contact with coordinate (x_1, y_1, z_1) and (x_2, y_2, z_2), the distance r between them can be described as:

$$r = \sqrt{r_{\mathrm{xy}}{}^2 + (z_1 - z_2)^2}, \tag{S5}$$

in which $r_{xy} = \sqrt{(x_1 - x_2)^2 + (y_1 - y_2)^2}$ is the distance in x-y plane. Considering that the action range of vdW forces is much smaller than the scale of the surfaces, the total potential between surfaces of unit area can be calculated as[11]:

$$V_s(h) = \rho_1\rho_2 \int_{-\infty}^{0}\int_{h}^{\infty}\int_{0}^{\infty} V_{LJ}\, 2\pi r_{xy} dr_{xy}\, dz_1\, dz_2$$

$$= 4\varepsilon\rho_1\rho_2\pi\left(\frac{\sigma^{12}}{360h^8} - \frac{\sigma^6}{12h^2}\right), \quad \text{(S6)}$$

In which $\rho_1 = 1.14 \times 10^{29}\ \mathrm{m^{-3}}$ and $\rho_2 = 5.01 \times 10^{28}\ \mathrm{m^{-3}}$ are the atom number densities of carbon in graphite and silicon in substrate, h is the distance between two surfaces. The pressure of vdW interaction between graphite and silicon surfaces is:

$$P(h) = -\frac{dV_s(h)}{dh} = \varepsilon\rho_1\rho_2\pi\left(\frac{4\sigma^{12}}{45h^9} - \frac{2\sigma^6}{3h^3}\right). \quad \text{(S7)}$$

The curve of function $P(h)$ is shown as the blue line in Supplementary Fig. 12. The balance position marked as point A takes place when $h =$ 2.6 Å, and the max adhesive pressure $P_{max} = 860\mathrm{MPa}$ appears when $h =$ 3.1 Å, which is marked as point B. The adhesive pressure declines rapidly as h increases, and when the distance between surfaces is greater than 1 nm, the vdW interaction is negligible. The red line in Supplementary Fig. 12 is the approximation of vdW interaction used in the finite element methods, where the coordinates of points C and D are (5, 300) and (10,0). The straight line to the left of point A is the tangent of the function curve (blue line) at point A.

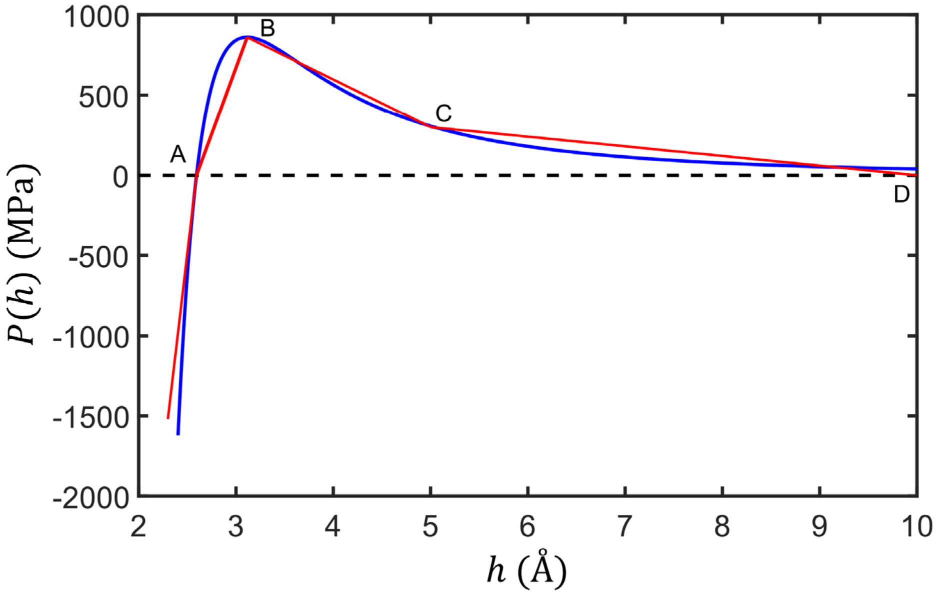

Supplementary Fig. 12 Pressure of van del Waals interaction between graphite and silicon substrate. Blue line is the vdW interaction derived from LJ potential (Eq. (S7)) between carbon and silicon atoms. The red line is the difference approximation curve used in the finite element method.

8.2 Mesh convergence analysis

In our FEM model in Fig. 3 in main text, we used the 8-node linear hexahedral solid element with reduced integration. The number of elements is 776900, which the average size of the elements is about 20nm. In order to analyze the mesh convergence of our model, we conducted simulations with different number of elements under the same setting, and extracted two critical parameters in the simulation results, namely the maximum stress $U_{\max}^{(\mathrm{Mises})}$ on the bottom surface of graphite flake (upper) and the minimum displacement in height $\Delta h_{\min}$ along the graphite flake edge (lower), as shown in the Supplementary Fig. 13. The results show that with the refinement of the mesh, the rate of change of the two characteristic parameters gradually decreases and tends to converge, and sufficient accuracy can be obtained under the number of elements we use.

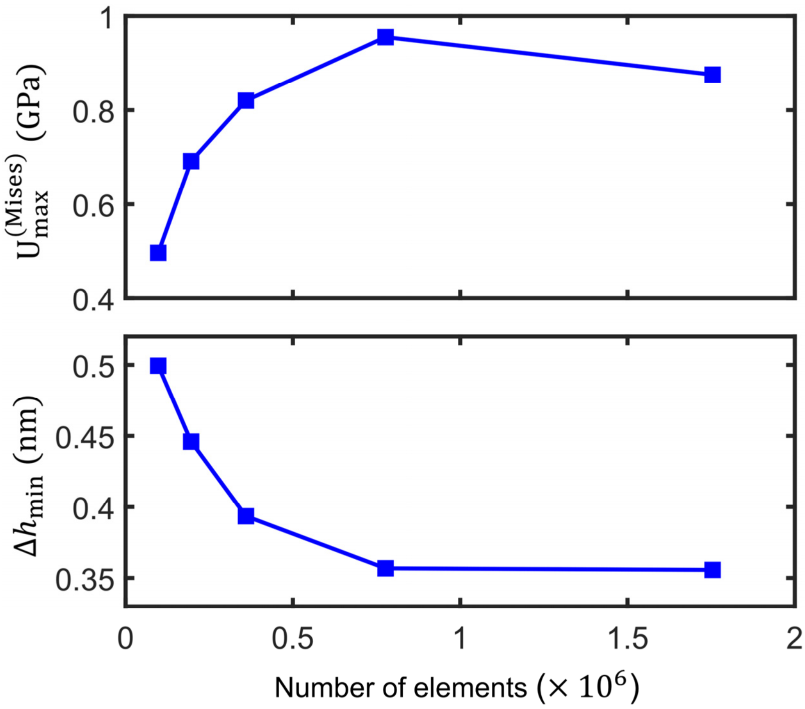

Supplementary Fig. 13 Mesh convergence analysis of our FEM model. The maximum Mises stress $U_{max}^{(Mises)}$ on the bottom surface of the graphite flake (upper) and the minimum displacement in height Δh_{min} along the graphite flake edge (lower) for different element numbers.

8.3 Simulation of graphite flake pressed on flat silicon surface

We performed comparative finite element method simulations of graphite flake pressed against a flat silicon substrate. The model is shown in Supplementary Fig. 14a, in which the flat silicon substrate has the same size and thickness as the nanostructured silicon substrate but no rough peaks, and the other simulation settings and boundary conditions are consistent with graphite flakes on nanostructured silicon (Fig. 3a in main text). The Mises stress distribution across the diagonal section is shown in Supplementary Fig. 14b. Compared to graphite flakes pressed on the surface of nanostructured silicon, there is also a normal stress concentration near the loading center, but no stress concentration around, and stress exists in the peripheral areas of the graphite flake and the flat silicon. We further plot the displacement in height (upper)

and Mises stress (lower) distribution along the bottom of the graphite flake pressed on flat silicon across the diagonal section, as shown in Supplementary Fig. 14c, which shows that the peripheral part of the graphite flake bottom on a flat silicon substrate remained in its original position (black dotted line) with no displacement in height under central loading. Therefore, the contact between graphite flake and flat silicon surface is continuously in the whole contact area and no warping of edges occurs.

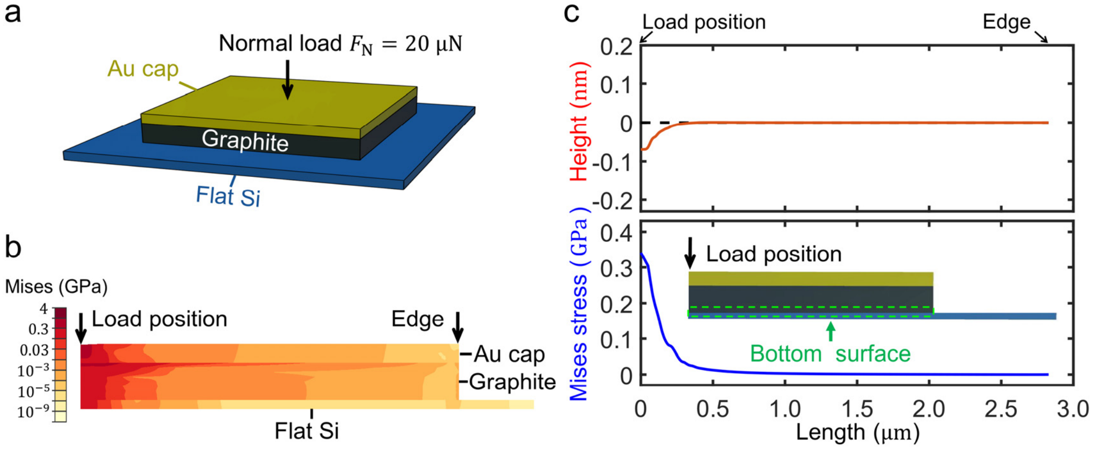

Supplementary Fig. 14 Comparative finite element method simulations of graphite flake pressed against a flat silicon substrate. a Schematic diagram of the finite element simulation model with flat silicon/graphite flake heterojunction, where the concentrated normal load of 20 μN is applied to the center of the graphite flake. **b** Mises stress distribution across the diagonal section under central normal loading. **c** Displacement in height (upper) and Mises stress (lower) distribution along the bottom interface of graphite flake across diagonal section (see illustration in the lower image), where the black dotted line in the upper image is the original position of the bottom interface of the graphite flake.

8.4 Calculation of the real contact area between graphite flakes and nanostructured silicon

For finite element method model shown in Fig. 3 of main text, the pressure distribution between the bottom surface of the graphite flake and nanostructured silicon substrate can be obtained as shown in Supplementary Fig. 15. The real contact area between graphite flake and nanostructured silicon surface (i.e., the location where the

pressure distribution is greater than zero) can be divided into two parts: One part is the center area of the bottom surface of the graphite flake, which is pressed on the nanostructured silicon under concentrated load; the other part is the contact area between the bottom surface of the graphite flake and some rough peaks close to the loading center.

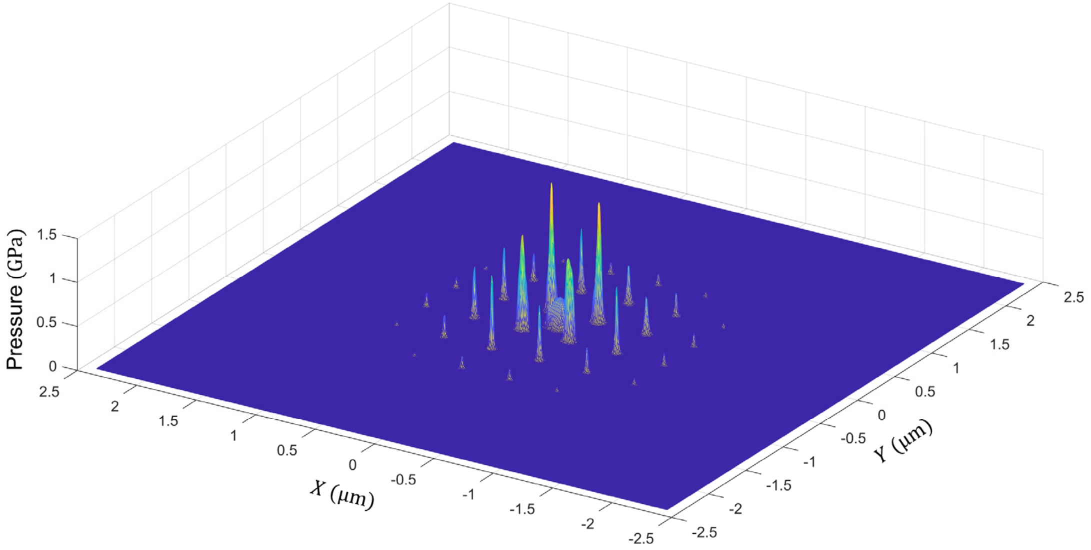

Supplementary Fig. 15 Simulated pressure distribution between the bottom surface of the graphite flake and nanostructured silicon substrate. Since there are tens of thousands of data points in the original data, it is difficult to draw graphs with software, so we use 600×600 difference points to draw graphs, resulting in asymmetric pressure distribution.

The contact area in the center area of the bottom surface of the graphite flake, which is pressed on the nanostructured silicon under concentrated load, can be approximated to a circle with radius $r = 0.124$ μm, and the contact area between the bottom surface of graphite flake and the 32 rough peaks which keep in contact under the normal load can be calculated through DMT model. According to the DMT model, the contact area is given by:

$$A = \pi \left(\frac{R}{K} (F_{\mathrm{N}} + 2\pi R\gamma) \right)^{\frac{2}{3}} \tag{S8}$$

where $R = 0.177\ \mu\text{m}$ is the curvature radius of the top of the rough peak, F_{N} is the normal force between the graphite flake and a single rough peak, $\gamma = 1.95 \times 10^{-2}\ \text{J/m}^2$ is the energy of adhesion which can be derived from the van del Waals interaction discussed above (Supplementary Section 8.1), and K is the reduced Young's modulus given as:

$$K^{-1} = \frac{3}{4}\left(\frac{1-\nu_{\text{g}}^2}{E_{\text{g}}} + \frac{1-\nu_{\text{s}}^2}{E_{\text{s}}}\right) \tag{S9}$$

where $E_{\text{g}} = 36.1\ \text{GPa}$ and $E_{\text{s}} = 112\ \text{GPa}$ are Young's modulus of the graphite in the contact direction and the silicon substrate, respectively; and $\nu_{\text{g}} = 0.17$ and $\nu_{\text{s}} = 0.28$ are the Poisson ratios of graphite and silicon, respectively. Combine Eq. (S8) and (S9), we calculated the real contact area between graphite flake and nanostructured silicon substrate under concentrated normal load to be $A = 6.23 \times 10^{-2}\mu\text{m}^2$.

8.5 Simulation of graphite flake pressed on nanostructured silicon surface with difference separation.

We performed finite element method (FEM) simulations of the warping of graphite flakes that placed on nanostructured silicon with different separation under central loading. The distance between nanoasperities gradually increases from 400nm in the experiment to 800nm with the same shape and size. Considering the cost, compared with the simulation work in the main text, this part of the simulation uses a coarser mesh division and model. The schematic diagram of the finite element simulation model is shown in Supplementary Fig. 16a. Several feature locations are marked with white dots in the figure, which are Center (center of the bottom surface), Middle (center of the edge), and Corner (corner of the edge). The feature lines connecting the white dots are diagonal of bottom surface (blue dotted line) and edge (yellow solid line). Supplementary Figs. 16b and c show the displacement in height distribution along the bottom interface of graphite flake across diagonal section (along the blue dotted line) and edge section (along the yellow solid line) under different separations d of the

nanostructures with a normal load of $20\ \mu\mathrm{N}$, respectively. Supplementary Figs. 16d and e represent the corner warpage height $\Delta h_{\mathrm{corner}}$ and center warpage height $\Delta h_{\mathrm{middle}}$ of the graphite flake edge under different normal forces, respectively. From the results we can conclude that the warpage height of the graphite flake edge decreases with the increase of the separation between the nanorough peaks (Supplementary Figs. 16c-e). When the nanorough peaks becomes sparse enough $(d > 600\ \mathrm{nm})$, even increasing the loading at the center of the graphite flake cannot warp the entire edge above the horizontal plane (Supplementary Figs. 16c and e). As a result, the part of the edge that sinks below the horizontal plane will interact with the nanorough peaks of the substrate (collisions, bonding and breaking, etc.), regenerating high friction and wear, thereby disrupting the robust SSL state. Therefore, controlling the size and separation of the nano-asperities is of great significance for the surface modification method in this work to achieve robust SSL.

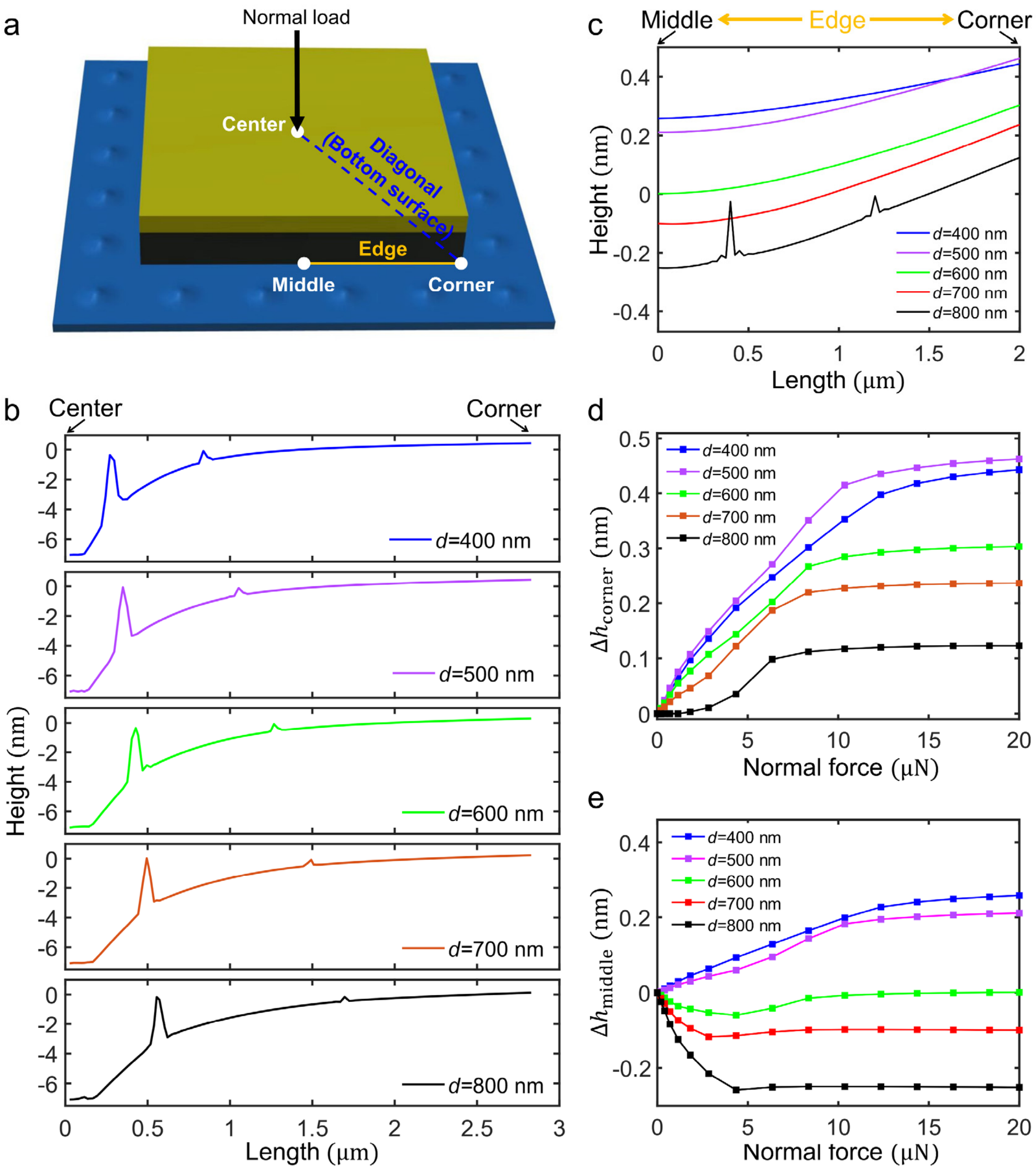

Supplementary Fig. 16 Simulation of graphite flakes under center loading on nanostructures with different separations. a Schematic diagram of the finite element simulation model, except that the separation of the nanostructure (that is, the distance d between rough peaks) is set to 400 nm 500 nm, 600 nm, 700 nm and 800 nm respectively, the other settings are consistent with Fig. 3 in the main text. Several feature locations are marked with white dots in the figure, which are Center (center of the bottom surface), Middle (center of the edge), and Corner (corner of the edge). The feature lines connecting the white dots are diagonal of bottom surface (blue dotted line) and edge (yellow solid line). **b** and **c** show the displacement in height distribution along

the bottom interface of graphite flake across diagonal section (along the blue dotted line in **a**) and edge section (along the yellow solid line in **a**) under different separations of the nanostructures with a normal load of 20 μN, respectively. **d** and **e** represent the corner warpage height Δh_{corner} and center warpage height Δh_{middle} of the graphite flake edge under different normal forces, respectively.

9. Layer-number-dependent molecular dynamics simulations of adhesion at the graphene/silicon interface

The van der Waals interaction between the graphite and the substrate is a short-ranged interaction. In our MD simulations, it is represented by Lennard-Jones potential, $V_{ij} = 4\varepsilon\left[\left(\sigma/r_{ij}\right)^{12} - \left(\sigma/r_{ij}\right)^{6}\right]$, where ε is the energy well depth, and σ is the characteristic length. Typically, the value of σ is 3 Å – comparable to the interlayer distance of graphite. According to the formula, V_{ij} becomes the high order small quantity ($< 10\%\ \varepsilon$) for $r_{ij} \gtrsim 2\sigma$; for $r_{ij} = 3\sigma$, the interaction becomes negligibly small, $V_{ij} \approx 0.5\%\ \varepsilon$. Thus, the use of bilayer graphene is believed to be accurate in estimating the adhesion.

To validate the discussion above, we performed additional simulations with 1 and 4 layers of graphene as shown in Supplementary Fig. 17. It can be seen from the simulation results that the system using bilayer graphene already gives the saturated value of adhesion.

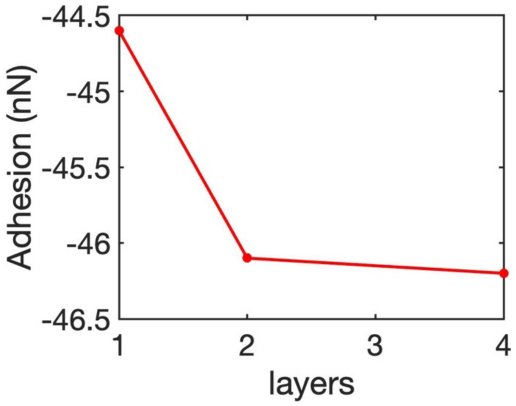

Supplementary Fig. 17 Molecular dynamics (MD) simulations of adhesion at graphene/silicon interfaces with different numbers of layers.

10. Analyse of the water film at the nanostructured surface

The contacts used in our experiments are composed of graphite and silicon surface. When exposed to ambient condition, e.g., the temperature of 25 ± 1 °C and relative humidity of 25± 1% as used in our experiment, the graphite will be covered with a water film of which the thickness is less than 1 nm[13]. For the silicon surfaces, there have also been detailed experimental measurements which shows that the thickness of the water film is also less than 1 nm[14-16]. To illustrate this point, we used a commercial instrument (DataPhysics-OCA 25) to measure the contact angle of water on the surface of the prepared nanostructured silicon under the same experimental environment. The test was done 4 times (at different positions), where the optical microscope observation during the test is shown in Supplementary Fig. 18. The measured contact angle and experimental parameters are shown in Supplementary Table 3, which presents a weak hydrophobicity (CA~ 92.30°- 101.48°). Therefore, such thickness, e.g., less than 2 nm in total, is much less than the typical height of 7nm for the nanostructures used in our experiments, so that the water film does not completely submerge the nanostructures, and the water film in the bottom region of the nanostructure surface does not directly contact the bottom surface of graphite flakes. In addition, even there was a thin water film covering the apex of the nanostructures, under a normal pressure of few hundreds MPa as used in our frictional test, the water film would be extruded from the contact between the graphite and the apex of the nanostructures, as it can sustain a normal load less than 20 MPa when confined between a nanoscale tip/pillar and graphite[17-19]. As a consequence, capillary condensation has no effects in the friction between the nanostructured silicon surface and graphite. For that between flat silicon surface and graphite, it may play a role, which however is beyond the scope of our present study.

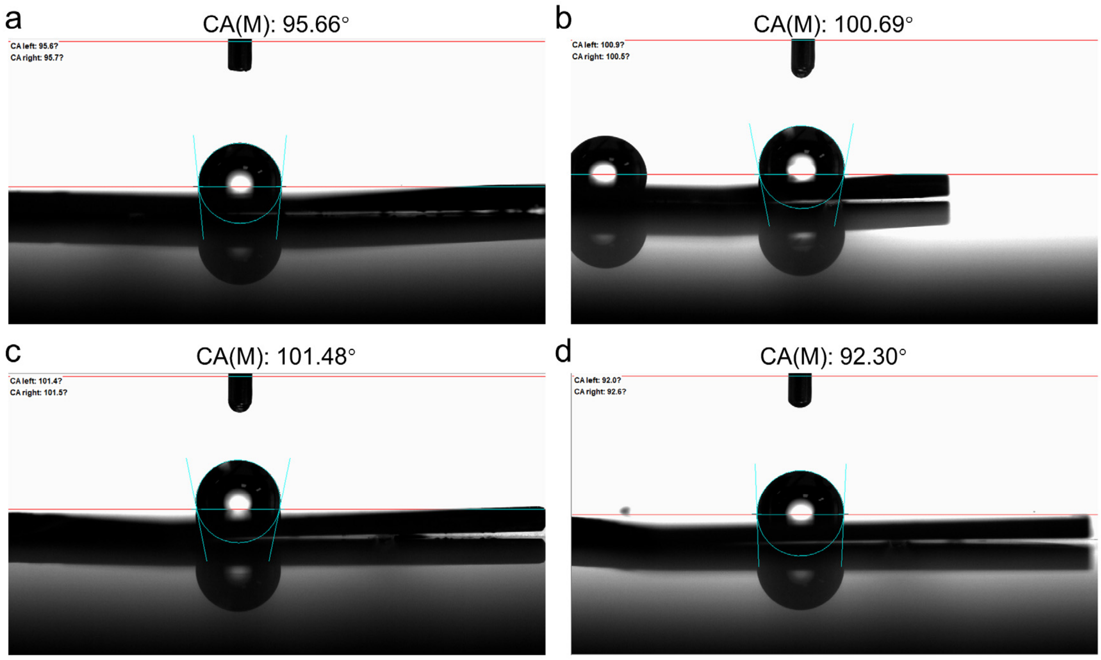

Supplementary Fig. 18 Contact angle measurements of water on prepared nanostructured silicon surface. a-d are the optical observations during the tests at different positions.

Supplementary Table 3 Measured contact angle and experimental parameters of water on prepared nanostructured silicon surface (temperature of 25 ± 1 °C and relative humidity of 25± 1%).

Experiments No.	CA(L)[°]	CA(R)[°]	CA(M)[°]	Drop volume [μL]	Contact diameter [mm]	Drop height[mm]
1	95.66	95.64	95.67	1.58	1.75	0.94
2	100.69	100.92	100.46	1.86	1.77	1.03
3	101.48	101.41	101.55	1.86	1.75	1.04
4	92.30	92.02	92.58	1.77	1.86	0.95

11. General surface modification methods for superlubric generators (SLGs) application scenarios

The surface modification method employed in this work provides a general surface modification method to achieve robust structural superlubric (SSL) state between

graphene flakes and non-vdW materials, which promote the general application of SSL technology. For example, as an application of SSL, several types of superlubric generators (SLGs) have been proposed[10,20]. SLG is designed to produce stable high current density and long-lifespan output under relative sliding through SSL technology, and the conversion efficiency is close to 100%[10,20]. Recently, the Schottky superlubric generator (S-SLG), that is, the sliding contact between micro-sized graphite flakes and n-type silicon in the SSL state, was proposed as a physical prototype of SLGs (Supplementary Fig. 19a), which can not only generate a stable and high current density of $\sim 210\mathrm{Am}^{-2}$ and power density of $\sim 7\ \mathrm{Wm}^{-2}$, but more importantly, achieve a long lifetime of at least 5,000 cycles while maintaining stable high electrical current density ($\sim 119\ \mathrm{Am}^{-2}$)[10].

However, the edges of graphite flake will cause high friction and wear with a certain probability, leading to the failure of the SLG, as shown in Supplementary Fig. 19a. Here, we demonstrate our surface modification method to optimize the original SLG to avoid high friction and wear, as shown in Supplementary Fig. 19b. The nanostructures are prepared on the n-Si surface, so that the edge of the graphite flake is warped under the central loading to avoid high friction and wear that may occur at graphite flake edges and form a robust SSL state. To promote SLG to large-scale batch applications, we can connect the centers of multiple graphite flakes placed on nanostructured n-Si with columnar Ni through sacrificial layer and electroplating methods to realize the center loading (inducing warpage curved edges) and integrated connections of surface-modified SLGs, as shown in Supplementary Fig. 19c. While the microfabrication process of such structure is under operation and takes time, we believe that with the mechanism revealed in our present manuscript, it is reasonable to believe that the friction and wear will greatly be reduced with the help of edge warping.

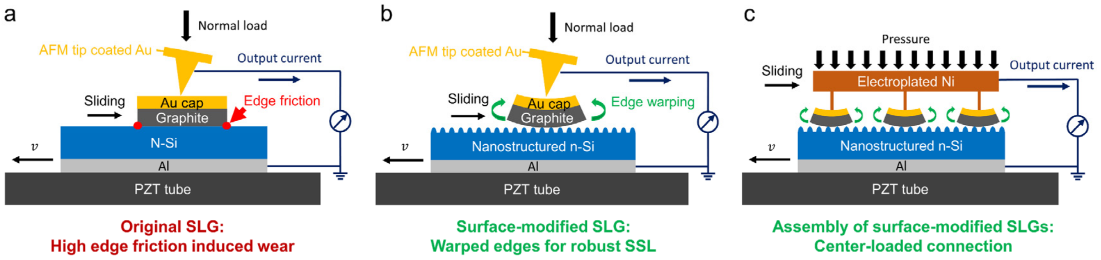

Supplementary Fig. 19 Optimized superlubric generators (SLGs) through surface modification. a Schematic diagram of original SLG, the graphite flake and n-Si form a structural superlubric (SSL) contact, which will produce stable high current density and long-lifespan output under relative sliding, but the edges will cause high friction and wear with a certain probability. **b** Schematic diagram of surface-modified SLG, nanostructures are prepared on the n-Si surface, so that the edge of the graphite flake is warped under the central loading to avoid high friction and wear that may occur at graphite flake edges and form a robust SSL state. **c** Assembly of surface-modified SLGs, the centers of multiple graphite flakes placed on nanostructured n-Si are connected with columnar nickel by sacrificial layer and electroplating methods, realizing the functions of center loading (inducing warped edges for robust SSL) and integrated connection to promote SLG towards large-scale batch application.

Reference

1 Lu, X. K., Yu, M. F., Huang, H. & Ruoff, R. S. Tailoring graphite with the goal of achieving single sheets. *Nanotechnology* **10**, 269-272, doi:10.1088/0957-4484/10/3/308 (1999).

2 Jensen, D. S. *et al.* Silicon (100)/SiO2 by XPS. *Surface Science Spectra* **20**, 36-42 (2013).

3 Girardeaux, C. & Pireaux, J. J. Analysis of polystyrene (PS) by XPS. *Surface Science Spectra* **4**, 130-133, doi:10.1116/1.1247812 (1996).

4 Bhatia, Q. S., Pan, D. H. & Koberstein, J. T. Preferential surface adsorption in miscible blends of polystyrene and poly (vinyl methyl ether). *Macromolecules* **21**, 2166-2175 (1988).

5 Zheng, Q. *et al.* Self-retracting motion of graphite microflakes. *Physical Review Letters* **100**, doi:10.1103/PhysRevLett.100.067205 (2008).

6 Wang, K., Qu, C., Wang, J., Quan, B. & Zheng, Q. Characterization of a microscale superlubric graphite interface. *Physical Review Letters* **125**, doi:10.1103/PhysRevLett.125.026101 (2020).

7 Sader, J. E., Chon, J. W. M. & Mulvaney, P. Calibration of rectangular atomic force microscope cantilevers. *Review of Scientific Instruments* **70**, 3967-3969, doi:10.1063/1.1150021 (1999).

8 Sader, J. E., Larson, I., Mulvaney, P. & White, L. R. Method for the calibration of atomic-force microscope cantilevers. *Review of Scientific Instruments* **66**, 3789-3798, doi:10.1063/1.1145439 (1995).

9 Li, Q., Kim, K. S. & Rydberg, A. Lateral force calibration of an atomic force microscope with a diamagnetic levitation spring system. *Review of Scientific Instruments* **77**, doi:10.1063/1.2209953 (2006).
10 Huang, X. *et al.* Microscale Schottky superlubric generator with high direct-current density and ultralong life. *Nature communications* **12**, 1-10 (2021).
11 Peng, D. *et al.* 100 km wear-free sliding achieved by microscale superlubric graphite/DLC heterojunctions under ambient conditions. *National Science Review* (2021).
12 Rappé, A. K., Casewit, C. J., Colwell, K., Goddard III, W. A. & Skiff, W. M. UFF, a full periodic table force field for molecular mechanics and molecular dynamics simulations. *J. Am. Chem. Soc.* **114**, 10024-10035 (1992).
13 Wang, K. *et al.* Structural superlubricity with a contaminant-rich interface. *Journal of the Mechanics and Physics of Solids* **169**, doi:10.1016/j.jmps.2022.105063 (2022).
14 Chen, L., He, X., Liu, H., Qian, L. & Kim, S. H. Water adsorption on hydrophilic and hydrophobic surfaces of silicon. *The Journal of Physical Chemistry C* **122**, 11385-11391 (2018).
15 Chen, L. *et al.* Dependence of water adsorption on the surface structure of silicon wafers aged under different environmental conditions. *Physical Chemistry Chemical Physics* **21**, 26041-26048 (2019).
16 Cao, P., Xu, K., Varghese, J. O. & Heath, J. R. The microscopic structure of adsorbed water on hydrophobic surfaces under ambient conditions. *Nano letters* **11**, 5581-5586 (2011).
17 Li, J., Cao, W., Li, J. & Ma, M. Fluorination to enhance superlubricity performance between self-assembled monolayer and graphite in water. *Journal of Colloid and Interface Science* **596**, 44-53 (2021).
18 Li, J., Cao, W., Li, J., Ma, M. & Luo, J. Molecular origin of superlubricity between graphene and a highly hydrophobic surface in water. *The journal of physical chemistry letters* **10**, 2978-2984 (2019).
19 Liu, B. *et al.* Negative friction coefficient in microscale graphite/mica layered heterojunctions. *Science Advances* **6**, eaaz6787 (2020).
20 Huang, X., Lin, L. & Zheng, Q. Theoretical study of superlubric nanogenerators with superb performances. *Nano Energy*, 104494 (2020).
